# The Anti-Cancer Effect of Quercetin: Molecular Implications in Cancer Metabolism

**DOI:** 10.3390/ijms20133177

**Published:** 2019-06-28

**Authors:** Marjorie Reyes-Farias, Catalina Carrasco-Pozo

**Affiliations:** 1 Department of Endocrinology and Nutrition, Germans Trias i Pujol Research Institute, 08916 Barcelona, Spain; 2 Discovery Biology, Griffith Institute for Drug Discovery, Griffith University, Nathan, QLD 4111, Australia

**Keywords:** quercetin, cancer, glycolysis, mitochondrial function, PI3K/Akt pathway

## Abstract

Cancer is a problem with worldwide importance and is the second leading cause of death globally. Cancer cells reprogram their metabolism to support their uncontrolled expansion by increasing biomass (anabolic metabolism—glycolysis) at the expense of their energy (bioenergetics-mitochondrial function) requirements. In this aspect, metabolic reprogramming stands out as a key biological process in understanding the conversion of a normal cell into a neoplastic precursor. Quercetin is the major representative of the flavonoid subclass of flavonols. Quercetin is ubiquitously present in fruits and vegetables, being one of the most common dietary flavonols in the western diet. The anti-cancer effects of quercetin include its ability to promote the loss of cell viability, apoptosis and autophagy through the modulation of PI3K/Akt/mTOR, Wnt/β-catenin, and MAPK/ERK1/2 pathways. In this review, we discuss the role of quercetin in cancer metabolism, addressing specifically its ability to target molecular pathways involved in glucose metabolism and mitochondrial function.

Cancer is a problem with worldwide importance and is the second leading cause of death globally. In 2018, there were 18.1 million new cases of cancer and 9.6 million deaths caused by cancer disease [1]. This pathology causes a high economic impact on public and private health. For example, in the US, the total annual economic cost due to cancer was US$ 1.16 trillon in 2010 [2]. Cancer Tomorrow has predicted that in 2040 there will be a 63.1% increase in new cases of all types of cancer (29.5 million more) compared to 2018 [3].

The types of cancer with the highest incidence worldwide are lung (11.6% of total), breast (11.6%), colorectal (10.2%), prostate (7.1%), and stomach cancer (5.7%). The highest rates of world deaths are associated with lung (18.4%), colorectal (9.2%), stomach (8.2%), liver (8.2%), and breast cancer (6.6%). Regarding incidence, mortality, and 5 year prevalence by region, Asia has the highest levels (48.4%, 57.3%, and 39.7%, respectively), followed by Europe (23.4%, 20.3%, and 27.7%, respectively) and North America (13.2%, 7.3%, and 18.5%, respectively) [2]. Although high incidence rates are associated with high-income countries [4], 70% of deaths from cancer occur in low- and middle-income countries [5]. Considering these elevated statistics, it is relevant to work on therapeutic strategies to prevent and treat cancer. In this sense, it has been reported that dietary natural products could be a cost-effective alternative, as they can be regularly delivered by the diet, to promote cancer prevention or complement pharmacological therapies in cancer treatment.

## 1. Aerobic Glycolysis as Energetic Source in Cancer Cells

Deregulation of cell proliferation is a prerequisite for carcinogenesis. A single genetically altered cell causes abnormal proliferation, which leads to the outgrowth of a population of clonally derived tumor cells. However, for this to be possible, the production of biomass and energy are key points to allow and sustain uncontrolled cell expansion [6]. As a tumor is characterized by limited nutrient availability, cancer cells undergo competitions for nutrients with other types of cells, which are part of their microenvironments [7,8]. Therefore, uncontrollable cell proliferation and tumor formation induce a change in metabolic requirements, as well as in nutrient and oxygen cellular availability [9,10], which promotes metabolic reprogramming [11,12,13]. Because the tumor environment is limited in nutrients, it is important that these cells have a mechanism to support energy demand for proliferation and cell growth. Likewise, it has been described that a change in glucose metabolism called the “Warbug Effect” occurs in cancer cells [14,15]. Basically, in cancer, cells favour metabolism via aerobic glycolysis with the accumulation of lactate, in contrast to the more energetic, efficient oxidative phosphorylation that occurs in normal cells [6]. This switch in cell metabolism is beneficial for cancer progression and resistance to therapy [6,16,17]. Aerobic glycolysis is an inefficient way to produce ATP because it only generates 2 ATPs per molecule of glucose, compared with oxidative phosphorylation, which generates 36 ATPs per molecule [18]. However, aerobic glycolysis remains the preferred metabolic mechanism for cancer cells because biomass production is prioritized over energy production [7,19]. In this sense, in cancer cells, glycolysis generates high levels of carbon-rich metabolic intermediates that could be utilized as precursors for de novo synthesis of nucleotides, lipids, or amino acids [20,21]. Specifically, glucose-6-phosphate and fructose-6-phosphate, glyceraldehyde-3-phosphate, and 3-phosphoglycerate are glycolytic intermediates that are substrates for nucleotide, aminoacid and lipid biosynthesis [21].

## 2. Molecular Network That Underlies Metabolic Reprogramming in Cancer

In cancer cells, metabolic reprogramming is regulated by several pathways, including the phosphoinositide 3-kinase/protein kinase B (PI3K/Akt), which promotes an increased glucose uptake and glycolysis [22,23]. PI3K transduces the signal across messengers that activate Akt, and thus completely active, the PI3K/Akt pathway regulates cell angiogenesis, metabolism, growth, proliferation, survival, protein synthesis, transcription and apoptosis [24,25]. Akt is involved in pathways that control the availability of nutrients by acting through AMP-activated protein kinase (AMPK). AMPK controls glucose and lipid metabolism by sensing changes in nutrient and extracellular energy levels [26]. AMPK is an energy sensor that is activated when intracellular levels of AMP are high. Stresses that stimulate ATP consumption or inhibit ATP production result in an increased AMP:ATP ratio, promoting AMPK activation [27]. Interestingly, AMPK can exert pro- or anti-tumor effects based on the metabolic context. AMPK activation promotes net ATP conservation by activating pathways of catabolic metabolism and inhibiting anabolic processes that consume ATP [27]. Under metabolic stress, AMPK can support tumorigenesis by promoting metabolic plasticity through stimulating alternative metabolic pathways, including mitophagy and fatty acid oxidation. However, AMPK activation can also inhibit cell growth by inducing a p53-mediated cell cycle arrest, thereby downregulating mammalian target of rapamycin C1 (mTORC1) activity [27]. Akt activation increases glycolysis by promoting hexokinase 2, which directly interact with the mitochondrial pore to prevent the release of apoptotic proteins [28]. In addition, Akt activates mTORC1/2 [29], which regulates the downstream signaling of protein and lipid synthesis, metabolism, cell survival and apoptosis [29,30,31]. The activated mTOR directly or indirectly stimulates metabolic reprogramming by activating key transcription factors, such as c-Myc, cyclin D, and hypoxia-inducible transcription factor 1 alpha (HIF-1α), even under normoxic conditions [32].

Major oncogenes, such as *c-Myc*, and *HIF-1α*, are reported to be master inducers of cancer glycolysis through direct or indirect transactivation of cancer glycolytic genes [33]. In normal cells, *c-Myc* expression is induced upon growth factor stimulation and involved in many biological processes, including proliferation, cell cycle progression, cell growth, metabolism, angiogenesis, differentiation, cell adhesion, and mobility [34]. However, in cancer cells, *c-Myc* expression promotes energy production and anabolic processes, which are required for rapid proliferation, independent of growth factor stimulation. *c-Myc* influences glucose metabolism by up-regulating the gene expression of glucose transporters and many glycolytic enzymes, such as pyruvate kinase, as well as lactate dehydrogenase A (*LDH-A*) [34]. *HIF-1α* is involved in adaptive responses upon reduced oxygen availability and is a transcription factor that controls glycolytic gene expression. In fact, HIF-1α is the primary driver of increased glycolysis and lactate production during hypoxia [35]. In addition, HIF-1α up-regulates the vascular endothelial growth factor (*VEGF*), erythropoietin (*EPO*), glucose transporter 1 (*GLUT1*), and glycolytic enzymes genes under normal O_2_ conditions [36,37]. Besides promoting a high glycolytic rate, *HIF-1α* activation also inhibits oxidative phosphorylation by up-regulating genes, such as pyruvate dehydrogenase kinase 1 (*PDK1*) and *LDH-A*. In this way, the entrance of pyruvate into the TCA cycle is decreased [38]. Due to the role of HIF-1α in aerobic glycolysis and mitochondrial function, it has been proposed that HIF-1α inhibition could be a therapeutic strategy to treat cancer [37,38,39,40].

## 3. Role of Quercetin as an Anti-Cancer Agent—Molecular Implications

Quercetin (QUE; 3,5,7,3′,4′-pentahydroxyflavone) is the major representative of the flavonoid subclass of flavonols. Quercetin is ubiquitously present in fruits and vegetables, being one of the most common dietary flavonols in the western diet. According to the US Department of Health and Human Services, the average daily intake of QUE in humans is about 25 mg [41], a value which is also supported by French and Finnish studies [42,43]. Onions are among the foods particularly high in this flavanol, being 0.03–0.28 mg/100 g of the fresh weight (FW) of white and yellow onions, with red onion varieties exhibiting the highest content (around 1.31 mg/100 g FW) [44,45,46]. Quercetin has been reported to exert a wide range of biological effects, including antioxidant, anticarcinogenic, anti-inflammatory, anti-diabetic and antimicrobial activities [47,48,49,50,51,52,53]. In plants, QUE is mostly found in the form of glycosides [54]. β-glycosidases in the intestine catalyze the hydrolysis of glycosidic bonds before being absorbed into the enterocytes [54,55]. Here, they are metabolized into quercetin conjugates [54]. The bioavailability of QUE is low, which affects its systemic effects and may explain the differences between the QUE effects found *in vitro* and *in vivo* [54,55].

Quercetin is a flavonoid with high potential in oncology due to its chemopreventive effects evidenced in *in vitro* and *in vivo* models. Quercetin elicits biphasic, dose-dependent effects. At low concentrations, QUE acts as an antioxidant, and thus elicits chemopreventive effects, but at high concentrations, QUE functions as a pro-oxidant and may, therefore, elicit chemotherapeutic effects [56]. Quercetin’s anti-cancer effects rely on its ability to reduce proliferation, induce apoptosis, cause cell cycle arrest and inhibit mitotic processes by modulating cyclins, pro-apototic, PI3K/Akt and mitogen-activated protein kinase (MAPK) molecular pathways.

### 3.1. Effect in Cell Proliferation

It has been documented that QUE is able to inhibit *in vitro* proliferation of several cancer cell lines [57,58,59,60,61,62,63,64]. Quercetin (0–200 µM, 24 h incubation [61] and 0–300 μΜ, 5 days incubation [58]) inhibited the cell viability of colon cancer cell lines HCT-15 and RKO [61] and inhibited the proliferation of the breast cancer cell lines MCF-7, MDA-MB-231, HBL100 and BT549, and the ovarian cancer cell lines, OVCAR5, TOV112D, OVCAR3 and CAOV3 [58,64]. Quercetin also reduced the cell viability and growth of B-lymphoma (PEL, an aggressive B cell lymphoma cell) cells BC3, BCBL1 and BC1 in a wide range of concentrations (12 to 100 μM, 24 h incubation) but had no cytotoxic effect in normal B lymphocytes [62]. Quercetin at 100 and 150 μM (added every 24 h for 72 h) reduced prostate cancer PC3 cell proliferation to 83% and 64.17%, without causing cytotoxicity [63]. Quercetin, at a physiologically relevant concentration (0–10 μM, 4 days incubation), inhibited the proliferation of the breast cancer cell lines SK-Br3 and MDA-MB-453 in a dose dependent manner [59]. Although a low dose of QUE showed a mild cytotoxic effect, cell cycle arrest in the G1 phase was the main cause of anti-proliferation effect of QUE. This was mediated by down-regulating cyclin B1 and cyclin-dependent kinase 1 (CDK1), essential components of G2/M cell cycle progression and by inducing phosphorylation of the retinoblastoma tumor suppressor protein, pRb [59]. Hypophosphorylated Rb binds to and sequesters the transcription factor E2F1, an essential transcriptional factor required for the expression of cell proliferation-associated genes, resulting in cell cycle arrest at the G1 phase [65]. Quercetin also induced p21, a cyclin-dependent kinase (CDK) inhibitor, by inducing mild DNA damage and Chk2 activation [59]. Quercetin (50–130 µM) inhibited proliferation and increased the levels of the pro-apoptotic biomarker survivin in SKOV-3 ovarian cancer cells [57] and MCF-7 breast cancer cells [60], in a time- and dose-dependent manner. At a high concentration, QUE also inhibited cell cycle progression from G0/G1 to G2/M [57,60]. Quercetin displayed strong anti-mitotic activity by decreasing the activity of several kinases involved in the control of mitotic processes by more than 80%, such as Aurora kinases A and B, MET kinase, NIMA-related kinases (NEK4 and NEK9), PAKs (p21-activated kinases) and platelet-derived growth factor (PDGF) [66]. Interestingly, QUE exerts this effect at a low concentration (2 μM), which represents less than 10% of its IC_50_ growth-inhibitory concentration, as calculated from the average of eight distinct cancer cell lines (human non-small cell lung cancer, melanoma, glioblastoma, colon cancer, breast and prostate cancer cell lines, and the mouse melanoma cancer cell line) [66].

### 3.2. Effect on Oxidative Stress

A disturbance in the balance between the production of reactive oxygen species (ROS) and an organism’s antioxidant defense system leads to accumulation of ROS causing oxidative stress. Oxidative stress has been linked to the hyper-activation of signaling pathways for cell survival, proliferation and migration, as well as metabolic adaptations of tumor microenvironment [67]. ROS mediated signaling, leading to the activation of the PI3K/Akt pathway, plays an important role in the development of cancer [68]. Pretreatment with 300 µM QUE suppressed the increase of H_2_O_2_-induced ROS and significantly down-regulated the phosphorylation of Akt, PDK1, Bcl-2-associated death promoter (BAD) and the level of the tumor necrosis factor receptor 1 (TNFR1). Moreover, QUE increased the level of PTEN in H_2_O_2_ treated Dalton’s lymphoma ascite (DLA) cells [69]. As QUE prevented the alterations induced by H_2_O_2_ similar to the PI3k inhibitor PI-103, it is suggested that QUE attenuates PI3K/Akt pathway with a similar mechanism to PI-103 [69].

### 3.3. Effect in Autophagy and Apoptosis

In addition, several studies show that the anti-cancer effects of QUE are related to its ability to induce autophagy and apoptosis in cancer cells and xenograph models [62,64,70,71,72,73,74]. Quercetin, at 120 µΜ, increased the apoptotic rate (increased the exposure of phosphatidylserine on the outer leaflet of the plasma membrane) of the mouse colon carcinoma CT-26 cell line, androgen-sensitive prostate cancer LNCaP cell line, human T lymphoblast MOLT-4 (acute lymphoblastic leukemia), and human B lymphoblast Raji (Burkitt’s lymphoma) [70]. Quercetin increased the number of cells in the sub-G1 phase, nuclei fragmentation, activation of caspase-3 and caspase-9, and degradation of the poly(ADP-ribose) polymerase protein. Quercetin also reduced the mitochondrial membrane potential in U373MG malignant glioma cells. Quercetin induced intrinsic apoptosis through the activation of JNK and the increase in p53 expression and translocation to the mitochondria. It has been proposed that QUE induced protective autophagy in U373MG cells, as pretreatment with chloroquine, an autophagy inhibitor, strongly augmented apoptosis in these cells [71]. Quercetin also has the ability to induce protective autophagy in gastric and breast cancer cells by inactivating the Akt-mTOR pathway [64,74] and HIF-1α signaling [74]. Quercetin (12 to 100 μM, 24 h incubation) has also been shown to induce protective autophagy in PEL cells, increasing the cytotoxic effect of bortezomib, a proteasomal inhibitor. Quercetin also increased G1 phase cell and induced apoptosis in these cells, promoting poly (ADP-ribose) polymerase (PARP) cleavage and nuclear fragmentation. Additionally, QUE inhibited the phosphorylation of mTOR and Akt^ser473^, promoted glycogen synthase kinase 3 (GSK-3) dephosphorylation/activation, downregulated the expression of prosurvival cellular proteins such as cellular FLICE-inhibitory protein (c-FLIP), cyclin D1, and c-Myc, and induced the degradation of β-catenin. Moreover, QUE decreased the release of interleukin-6 (IL-6) and IL-10, and the phosphorylation/activation of the signal transducer and activator of transcription 3 (STAT3)^Tyr705/Ser727^ [62]. These two cytokines are involved in PI3K/Akt/mTOR and STAT3 activation. PI3K/Akt/mTOR signaling is linked to the Wnt/β-catenin pathway, since Akt phosphorylates GSK-3, leading to its inactivation and, consequently, to β-catenin accumulation [75]. These findings suggest that QUE induces cell death by inhibiting PI3K/Akt/mTOR and STAT3 pathways in PEL cells [62]. Quercetin has been shown to promote mitochondrial-mediated apoptosis and protective autophagy at the same time, by inducing endoplasmic reticulum stress via the p-STAT3/Bcl-2 axis in CAOV3 human ovarian cancer cells [72]. Moreover, the autophagy scavenger 3-methyladenine was shown to enhance QUE (80 mg/kg i.p. twice a week, for 4 weeks) anti-cancer effects in an ovarian cancer mice xenograft model, potentiating tumor size reduction and caspase-3 activation [72]. Quercetin (6 doses of 1 mg/kg orally, every third day) increased the DNA fragmentation in tumor tissues and reduced the tumor volume of breast adenocarcinoma in mice [73]. Quercetin (50 mg/kg i.p. twice daily for a month) reduced tumor size and decreased the level of the autophagy marker protein Beclin1 and the rate of p-Akt/Akt in tumor tissues in a breast cancer xenograft mouse model [64].

### 3.4. Effect of Quercetin as Co-Adjuvant Agent in Anti-Cancer Therapy

In combination with chemotherapy and radiotherapy, QUE acts in synergy to increase treatment sensitivity while protecting healthy cells from adverse effects. In combined treatments with QUE and SN-38 (active metabolite of irinotecan, inhibitor of DNA topoisomerase I), the cell viability, percentage of apoptosis, and β-catenin protein levels were comparable to those after treatment with high-dose SN-38 alone in the AGS human gastric adenocarcinoma cell line [76]. The combination of QUE and irinotecan had a stronger inhibitory effect on tumor growth than irinotecan alone, which was associated with a reduced gene expression of cyclooxygenase-2 and epithelial-mesenchymal transition-related markers. Furthermore, a combination with QUE also reduced the concentration of angiogenesis-associated factors (VEGF-A and VEGF-receptor 2) and percentage of Tie2-expressing monocytes in the AGS (human gastric cancer cell line) xenograft mouse model [76]. Quercetin and midkine (MK) siRNA significantly suppressed the survival of PC3 androgen independent prostate cancer cells, androgen dependent LNCaP prostate cancer cells, and CD44+/CD133+ stem cell [77]. The combined use of the two agents further reduced cell proliferation compared to the individual use of each [77]. Quercetin co-administration significantly enhanced midkine siRNA (MKsi) treatment efficacy by increasing the expression of caspase 3, inhibiting the PI3K/Akt, MAPK/ERK1/2 (extracellular signal-regulated kinase 1/2) and nuclear factor kappa B (NF-κB) signaling pathways, as well as suppressing the ATP-binding cassette subfamily G member 2 (ABCG2) protein expression in CD44+/CD133+ cells [77]. Midkine is a heparin-binding growth factor, which is overexpressed in various cancer types compared to healthy controls. An antisense oligonucleotide and siRNA targeting the MK suppress tumor formation *in vitro* as well as in nude mice [78,79].

Quercetin has been shown to inhibit 12-O-tetradecanoylphorbol-13-acetate (TPA)-induced transformation of JB6 promotion-sensitive mouse skin epidermal (JB6 P+) cells by inhibiting, dose dependently, the activation of the activator protein-1 (AP-1) and NF-κB induced by TPA [80]. These transcription factor molecules, AP-1 and NF-κB, are involved in the neoplastic transformation and development of cancer and are regulated by upstream kinases, including MAPKs signaling pathways [81,82]. In addition, QUE inhibited mitogen-activated protein kinase kinase (MEK1) and Raf1 kinase activities, as well as the subsequently attenuated TPA-induced phosphorylation of ERK/p90 ribosomal S6 kinase in JB6 P cells [80]. Interestingly, in this study, QUE has shown itself to be a more potent inhibitor of MEK1 kinase activity than PD098059, a well-known MEK1 inhibitor [80]. MEK1 is an important downstream component of oncogenic RAS signaling. Thus, it is a potential target for disrupting MAPK signaling. In the distal colon mucosa of rats supplemented with QUE (a 10 g/kg diet for 11 weeks), the MAPK pathway was downregulated and tumor suppressor genes, such as PTEN, Tp53, and Msh2, were upregulated [83].

### 3.5. Anti-Cancer Effect of Quercetin Derivatives

Replacement of OH groups with O-methylated (OMe) moieties can enhance the metabolic stability of flavones while retaining antiproliferative potency [84]. OMe QUE analogs have been shown to inhibit cell proliferation after 72 h incubation with human lung cancers (A549, H157, H460, 1792, 1944), as well as melanoma (M14), cervical (Hela), neck and head (M4E), and human breast (SKBR) cancer cells. 4′-O-methylquercetin, 7-O-methylquercetin and 4′,7-dimethoxyquercetin were more potent in inhibiting cancer cell growth than QUE in A549, H157, H460, 1792, 1944, M14, SKBR, and Hela cells, indicating that the methylation at the 4′ and/or 7 positions improved activity [84]. Although 3-O-methylquercetin showed less antiproliferative potency compared to QUE, methylation of the OH groups at the 3 and 7 positions resulted in the most potent compound (3,7-O-dimethylquercetin), with an IC_50_ value of 0.46 μM against 1944 cells. This compound also showed potent cancer cell growth inhibition for A549, H157, H460, 1792, Hop62 and 1299 cells [84]. These findings suggest that methylation of the OH groups of QUE could represent potential lead compounds for novel anti-cancer agents.

Quercetin-6-C-β-d-glucopyranoside (QCG) is a cis-glycoside natural analog of QUE [85]. It inhibits prostate cancer PC-3, DU-145 and LNCaP cell proliferation by arresting cells at the G0/G1 phase of the cell cycle and induces apoptosis. QCG inhibits ROS generation and Akt/mTOR cell survival pathways [85]. Quercetin-3′-sulfate is the major metabolite of QUE found in human blood plasma and has been shown to decrease glucose and ATP levels in HepG2 cells, probably by interfering with GLUT2-mediated glucose uptake [86]. This metabolite could contribute to the systemic anti-cancer effect reported for QUE in *in vivo* models.

## 4. Anti-Cancer Effect of Quercetin by Targeting Key Molecular Factors in Anabolic Metabolism

Normal cells primarily use the oxidative phosphorylation for the production of energy and rely on glycolysis only when their oxygen supply is limited. In contrast, cancer cells frequently utilize glycolysis even in the presence of sufficient amounts of oxygen [6]. Recently, it has been proposed that in malignant cells, the metabolic shift to glycolysis is induced by their greater need for glucose metabolites (used for biomass production), instead of any specific impairment of the mitochondrial respiratory function [87]. From the perspective of biomass production, inhibiting glycolysis seems to be a key strategy to prevent and/or treat cancer. However, by including the bioenergetics perspective, inhibiting both glycolytic and mitochondrial pathways for ATP production could be a more efficient approach to kill cancer cells. Although multiple glycolytic inhibitors and mitochondria-targeted compounds have been developed, only a few have progressed to clinical trials, due to selectivity, efficacy and safety limitations [88,89]. More effort is needed in identifying compounds that target cancer metabolism. In this regard, QUE has been reported to target both glycolysis and mitochondrial functions [58,61,64,90,91,92,93,94,95,96,97,98].

Quercetin (26.5–50 μΜ) decreased glucose and lactate production in breast cancer HBL100 [58], MBA-MB-231 and MCF-7 cells [64], and ascites tumor cells [93,94,95], indicating that QUE inhibits glycolysis in these cells. Quercetin tested at the IC_50_ values (121.9 and 142.7 µM, determined by 24 h colon cancer cell proliferation, HCT-15, and RKO, respectively) decreased glucose consumption and lactate production time-dependently (4–10 h) in HCT-15 cells. However, in RKO cells, only the lactate production was affected and only after 4 h incubation [61]. Furthermore, pre-treatment with QUE at IC_50_ potentiated the inhibitory effect of 5-fluorouracil in the glycolytic metabolism of HCT-15 and RKO cells [61]. It is suggested that QUE alters glucose metabolism by inhibiting monocarboxylate transporter (MCT) activity [97,99]. These transporters excrete the glycolytic end-products, lactate and H+, and prevent the intracellular acidification-induced cell death of highly glycolytic cancer cells [96]. Quercetin dose- and time-dependently (25–150 μM, 24–72 h) reduced the extracellular acidification rate as a measurement of glycolysis in B164A5 murine melanoma cells. QUE, at a concentration of 9.24 µM, inhibited the activity of recombinant human pyruvate kinase isoenzyme M2 (PKM2), a key control point of glycolysis, by 50% [100]. Moreover, QUE decreased the protein levels of PKM2 and GLUT1 in breast cancer MDA-MB-231 and MCF-7 cells [64]. In acute myeloblastic leukemia HL60 cells, 50 μM of QUE effectively inhibited about 70% of the GLUT1 mediated methylglucose uptake [101]. Quercetin (50 mg/kg, i.p. twice daily for a month) reduced the PKM2 levels in the tumor tissue of a breast cancer xenograft mouse model [64]. This indicates that QUE may reduce glycolysis by targeting key enzymes of glucose metabolism and uptake. Interestingly, the QUE analogue isoquercitrin (quercetin 3-β-D-glucoside) increased the activity of recombinant PKM2 by 50% at a concentration of 1.34 µM [100]. Quercetin in foods is bound to sugars, mainly as β-glycosides, whose bioavailability is affected by their sugar moiety. Isoquercitrin’s bioavailability is only 5% of that observed with QUE, and in the gut, it is rapidly metabolized to QUE by β-glucosidases [102]. This suggests that the systemic and local (in the gut) effects of QUE (glucoside)-rich food are predominantly anti-carcinogenic by targeting glycolysis.

Quercetin (5–40 μM for 24 h incubation) concentration dependently decreased cell viability and caused very low apoptotic effect in acute myeloblastic leukemia HL60 cells [103]. However, when QUE was used in combination with 2-deoxy-d-glucose (2-DG), a glycolysis inhibitor, an additive effect was observed [103]. The additive cooperation between QUE and 2-DG was also observed in acute promyelocytic leukemia NB4 cells and leukemia monocytic THP-1 cells, although with lower efficacy than in HL60 cells. These cells showed different susceptibility to the effects of 2-DG and QUE. For instance, NB4 cells were more susceptible and THP-1 cells less susceptible to the cytotoxic action of 2-DG than HL60 cells, and THP-1 cells were more resistant to QUE. Although 2-DG has the ability to target glycolysis, its potential to be used as an anti-tumor drug is limited because 2-DG also stimulates the PI3K/Akt, MEK/ERK and insulin-like growth factor 1 receptor (IGF1R) pathways, promoting a survival signal in tumor cell lines [104]. In this regard, the association with QUE suppressed the effect of 2-DG in pro-survival pathways by preventing the stimulation of Akt phosphorylation in HL60 cells [103].

Additionally, QUE was characterized as mitochondria-targeting drugs in HL60 cells. The treatment with 20 µM QUE plus 5 mM 2-DG decreased the mitochondrial transmembrane potential (ΔΨm) and induced the inner mitochondrial membrane’s permeabilization (mIMP), a key step in triggering intrinsic apoptosis [103]. The ability of QUE to trigger intrinsic apoptosis by destabilizing mitochondrial membrane potential has been extensively reported for several cancer cell lines [105,106,107,108].

The effect of QUE in mitochondrial functions related to bioenergetics has been scarcely studied for cancer. Quercetin has been shown to induce mitochondrial dysfunction, reducing the basal and maximal respiration and the ATP-production liked to oxygen consumption in acute myeloblastic leukemia HL60 cells [90]. In fact, QUE is able to directly inhibit mitochondrial ATPase activity, with a 85% inhibition at 26.5 µM [93] or 50% inhibition at 36.4 µM [92].

The effect of QUE on glucose metabolism has also been evidenced *in vivo* and is associated to its ability to modulate the PI3K/Akt pathway. The administration of QUE (25–75 mg/kg, i.p. daily per 12 days) decreased, in a dose-dependent manner, the cell viability of the ascite cells of Dalton’s lymphoma mice, without causing liver toxicity [91]. In addition, QUE, in a dose-dependent manner, decreased mRNA expression and the activity of LDH-A, downregulated phosphorylation p85α without affecting its protein or mRNA levels, reduced Akt protein and mRNA levels, and increased p53 protein and mRNA levels in the ascite cells of Dalton’s lymphoma mice [91]. Quercetin (30 µM) also has been shown to decrease LDH-A protein levels in breast cancer, MDA-MB-231, and MCF-7 cells [64]. Decreased energy production due to insufficient LDH-A activity promoted by QUE caused poor survival in cancer cells. Decreased Akt levels, an essential mediator of PI3K signaling, and p85α phosphorylation, an essential component in PI3K activation, altered PI3K signaling in Dalton’s lymphoma mice treated with QUE [91]. These results indicate that QUE may inhibit glycolysis by downregulating the PI3K/Akt pathway. In fact, recently, QUE has been shown to directly inhibit PI3K enzymatic activity [98].

The acidic microenvironment produced by increased glycolysis in tumor cells provides a favorable microenvironment for tumor metastasis [109]. In fact, lactic acid activates VEGF, which promotes angiogenesis in tumors, triggers the exponential growth of cancer cells, and activates HIF-1α [18]. In addition, activation of the PI3K/Akt pathway in tumor cells can increase VEGF secretion, by the activation of both HIF-1 dependent and HIF-1 independent mechanisms [110]. PI3K/Akt pathway activation concludes in metalloproteinase (MMP) synthesis, promoting cell invasion [111]. As MMPs are involved in extracellular matrix remodeling, their role is crucial in the development of a favorable environment for the tumor’s metastatic process [112]. Treatment with 30 μM QUE suppressed the migration rate in wound-healing assays and in the trans-well invasion assay in breast cancer MCF-7 and MDA-MB-231 cells [64]. Consistently, QUE was able to reduce the protein levels of MMP-2, MMP-9, VEGF and mTOR, and p-Akt in breast cancer cell lines [64]. Quercetin (5 µM, 72 h incubation), acting as an MCT1 inhibitor, prevented cell invasion without influencing migration activity and the cellular expression of *MCT1* in human lung cancer A110L cells [99]. Moreover, QUE was also able to inhibit tumor metastasis and the progression of breast cancer in a xenograft mouse model by reducing the VEGF, PKM2, and p-Akt protein levels in tumor tissue [64]. These findings suggest that QUE exerts an inhibiting effect on cell mobility and glycolysis via the Akt-mTOR pathway in vitro and in vivo. The main effects of QUE in cancer, with an emphasis on cancer metabolism, are summarized in Table 1.

## 5. Clinical Trials Addressing Anti-Cancer Effect of Quercetin

According to the information available at ClinicalTrial.gov, there are several clinical trials studying the anti-cancer effect of QUE (Table 2). However, most of them, although completed, do not yet have available results [113,114,115,116]. The optimal dose of QUE (1 of 3 doses orally, twice a day for 6 to 10 weeks) that is effective in modulating biomarkers of colon epithelial cell turnover and, therefore, potentially inhibiting colon cancer development, was studied. However, although this trial was completed in 2006, no results have been revealed [113]. Quercetin contained in broccoli sprouts was also evaluated in the POUDER trial [114,117]. This trial specifically determined the feasibility of using a randomized controlled trial to test the application of freeze-dried broccoli sprouts rich in sulforaphane and QUE in patients receiving palliative chemotherapy with advanced pancreatic ductal adenocarcinoma (PDA) [114,117]. The POUDER trial was expected to transfer promising experimental and epidemiological data into a clinical pilot study to assess the effectiveness of broccoli sprout extracts in the treatment of advanced PDA. This study was completed in 2015, but its results have not been published so far [117].

In Oslo University Hospital, in collaboration with the University of Oslo, a dietary intervention study on patients with Follicular Lymphoma (FL) Stage III/IV was carried out to assess the ability of several dietary factors to induce apoptosis, inhibit cell proliferation, and modulate tumor cell infiltration in vivo. Grape juice containing resveratrol and QUE was evaluated among other dietary interventions. The last updated status from 2008 was “recruiting”. The study completion date was 2009, but no results have been published yet [115]. The effectiveness of QUE (500 mg QUE/day + vitamin C + folic acid + vitamin B3, for 6 months), in comparison with placebo, on the increase rate in the prostate-specific antigen (PSA) was carried out in a randomized controlled double-blind crossover trial by the University of Hohenheim, Germany [116]. As a secondary objective, the incidence of prostate cancer was evaluated, and the malondialdehyde and protein carbonyl levels, as indicators of oxidative status, were analyzed [116]. In the last update at ClinicalTrials.gov, from 2012, the status was “recruiting”. The study completion date was 2014. However, no results have been published yet.

In preclinical studies, green tea polyphenols have shown anti-cancer and cancer preventative effects in a number of malignancies [118]. In prostate cancer cells, QUE was found to enhance the anti-cancer effects of green tea [119]. Quercetin has been reported to increase the bioavailability of green tea polyphenols in in vitro and in vivo studies [120,121,122]. In the Jonsson Comprehensive Cancer Center, US, a clinical trial is currently evaluating the ability of QUE to enhance the uptake of green tea polyphenols in the prostate tissue of men taking green tea extract and undergoing radical prostatectomy [123]. The side effects of QUE in combination with green tea extract are also being assessed. This study will be completed in 2021 [123]. A clinical study to be completed in 2023 is evaluating the effect of QUE in delaying the development of Squamous Cell Carcinoma in patients with fanconi anemia. The need for potentially lethal treatment with chemotherapy and/or radiation therapy would be diminished or delayed with QUE treatment [124]. The efficacy of QUE will be determined in reducing the buccal micronuclei (an indicator of DNA damage, chromosomal instability, cell death, and the regenerative potential of human buccal mucosal tissue [125]) in post-hematopoietic cell transplantation patients with fanconi anemia. This study is being carried out at in the Children’s Hospital Medical Center, Cincinnati, US [124].

The promising anti-cancer effects of QUE, observed in in vitro and pre-clinical studies, established the basis to initiate several clinical trials evaluating the effectiveness of QUE to prevent and/or treat cancer and its associated complications. Unfortunately, the results of the completed trials have not been published yet. Thus, the anti-cancer effect of QUE in humans is still unclear.

## 6. Conclusions

Quercetin exerts its anti-cancer effects on cancer cells and tumors by modulating PI3K/Akt/mTOR, Wnt/β-catenin, and MAPK/ERK1/2 pathways. Quercetin promotes loss of cell viability, apoptosis and autophagy in cancer by reducing β-catenin and HIF-1α stabilization, inducing caspase-3 activation and inhibiting of Akt, mTOR, and ERK phosphorylation. Quercetin also prevents metastasis by reducing VEGF secretion and MMP levels. By interfering in PI3K/Akt/mTOR pathways, QUE exerts its metabolic effect on cancer, inhibiting key enzymes of glycolysis and glucose uptake. Quercetin also targets mitochondria in cancer, reducing bioenergetics and triggering intrinsic apoptosis. The effect of QUE on glucose metabolism and cellular energy production contributes to its effect on cell viability reduction, metastasis inhibition, and apoptosis induction in cancer cells.

Although, at this moment, there is no clinical evidence that QUE can prevent or treat cancer in humans, based on the studies discussed here, QUE could be useful as a diet supplement (in low doses) for cancer prevention and as a low-toxicity therapeutic molecule for cancer treatment.

## Abbreviations

2-DG2-DeoxyglucoseAktProtein Kinase BBaxbcl-2-like protein 4Bcl-2B-cell lymphoma 2 proteinCasp 3Caspase 3Casp 8Caspass 8Cox2Cyclooxygenase 2Cyt cCytochrome CERKExtracellular signal–regulated kinasesGLUT1Glucose transporter 1GSK3Glycogen synthase kinase 3LC3Microtubule-associated protein 1A/1B-light chain 3LC3-IILC3-phosphatidylethanolamine conjugateLDHLactate dehydrogenaseMAPKMitogen-activated protein kinasemIMPMitochondrial inner membrane permeabilizationMKsiMidkine silencerMMP-2Matrix metalloproteinase-2MMP-9Matrix metalloproteinase-9mTORmammalian Target of RapamycinNFκBNuclear factor kappa-light-chain-enhancer of activated B cellsp21Cip1Cyclin-dependent kinase inhibitor 1p27Kip1Cyclin-dependent kinase inhibitor 1Bp38Mitogen activated protein kinase p38p53Tumor protein p53p70-S6KRibosomal protein S6 kinase beta-1PI3KPhosphoinositide 3-kinasePKM2Pyruvate kinase isozyme M2PTENphosphatidylinositol-3,4,5-trisphosphate 3-phosphataseROSReactive oxygen speciesSurvivinbaculoviral inhibitor of apoptosis repeat-containing 5VEGFVascular endothelial growth factorVEGF-RReceptor of vascular endothelial growth factorΔΨmmitochondrial membrane potential

## Figures and Tables

**Table 1:** Anti-cancer effects of Quercetin (QUE).

Model	QUE Concentration	Anti-Cancer Effects	Reference
AGS cell line	6.25 to 100 μM	Reduced cell viability in a concentration-dependent manner; at dose 50 µM inhibited 50% growth. When added to SN-38, it improved the anti-proliferation effect of this compound, and increased apoptosis, acting synergistically with SN-38 in the modulation of GSK-3β/β-catenin signaling.	[76]
BALB/c nude mice injected with AGS cells	20 mg/kg BW	QUE alone (three times per week) or in combination with irinotecan (10 mg/kg once per week), promoted a significant reduction of tumor size at day 28, and also reduced tumor VEGF-R and VEGF-A levels, protein levels, and reduced COX-2 gene expression. This combination also decreased the TEM population.	[76]
MCF-7 and MDA-MB-231 cell lines	0 to 100 μM	Decreased viability (IC_50_ = 30 μM), increased autophagy, suppressed migration rate and reduced MMP-2, MMP-9 and VEGF protein levels. Also, suppressed glucose uptake, lactate production, and expression of PKM2, LDHA, and GLUT1. Similarly, QUE suppressed activation of AKT, mTOR, and p70-S6K.	[64]
Mice injected with MCF-7 cells	50 mg/kg BW	Inhibited tumor metastasis and progression of breast cancer. It also decreased VEGF, PKM2, and p-AKT levels in tumor tissue.	[64]
PC3 cells	1.78 to 100 μM	Inhibited survival in a dose- and time-dependent manner. At a concentration of 40 μM, QUE increased Cyt c, casp 3, casp 8, Bax, Bcl-2, p21_Cip1_, p27_Kip1_, and p53. QUE improved apoptotic effect of MKsi, increased casp 3 and decreased Survivin gene expression. QUE promoted the arrest in the G1 phase cells and decreased cells in the S-phase. QUE decreased the phosphorylation of PI3K, Akt, and ERK1/2. QUE decreased p38, NFκB, and Survivin protein levels and increased the PTEN expression. Combined with MKsi, QUE had a greater effect over ERK1/2, p38, NFκB, and Survivin.	[77]
B164A5 murine melanoma cells	150 μM at 72 h	Reduced OCR and ECAR.	[90]
BC3, BCBL1, and BC1 PEL cell lines.	12 to 100 μM	QUE for 24 h reduced cell survival and growth in a dose-dependent manner, without affecting normal B lymphocytes. QUE 50 μM increased apoptosis rate, increasing the G1 cell phase, PARP cleavage, and nuclear fragmentation/condensation. At this concentration, QUE also inhibited mTOR and Akt^ser473^ and promoted degradation of β-catenin.	[62]
MCF-7, MDA-MB-231, HBL100 and BT549 breast cancer cells, and OVCAR5, TOV112D, OVCAR3, CAOV3 ovarian cancer cells.	0.6 to 300 μΜ	Reduced cell proliferation concentration-dependently.	[58]
HBL100 cells	50 μΜ for 24 h	Increased intracellular accumulation of glucose and promoted lactate depletion into the culture media.	[58]
MCF-7 cells	300 μΜ for 48 h	Increased apoptosis by 25%.	[58]
HCT-15 and RKO cells	0 to 200µM	Inhibited cell proliferation, viability, and promoted apoptosis in a concentration dependent manner in cancer cells, but not in normal cells.	[61]
HCT-15 cells	142.7 µM (IC_50_)	Reduced glucose consumption and lactate production after 4 h incubation. QUE increased sensitization to 5-FU, improving its effects in glucose metabolism inhibition.	[61]
RKO cells	121.9 µM (IC_50_)	QUE increased sensitization to 5-FU, improving its effects in glucose metabolism inhibition.	[61]
DL mice	25 to 75 mg/kg BW	Decreased cell viability, mRNA expression and activity of LDH-A, in a dose-dependent manner, without generating liver toxicity. QUE downregulated p85a phosphorylation and Akt gene/protein expression and up-regulated mRNA expression of p53.	[91]
Ehrlich ascites tumor cells	26.5 µM	Inhibited lactate production by 78% and of Na^+^-K^+^-ATPase by 85%.	[93]
Ehrlich ascites tumor cells	13.25 to 66.17 µM	At a concentration of 26.5 µM, and after 10 min of treatment, QUE caused 50% inhibition in Na^+^-K^+^-ATPase activity. QUE inhibited aerobic glycolysis and oxidative phosphorylation in a concentration-dependent manner.	[92,126]
Ascites tumor cells	33.09 µM	Inhibited glycolysis and protein synthesis.	[94]
Rat thymocytes	25 µM	Prevented glucose uptake induced by mitogenic stimulus.	[127]
Ascites tumor cells	0.1µg/mg protein	Inhibited lactate efflux by 50%, increasing internal lactate concentration and decreasing intracellular pH.	[128]
HL60 cells	5 to 40 μM	QUE, and in combination with 2-DG, induced caspase-dependent late apoptosis, decrease of mitochondrial membrane potential and induction of mIMP, and attenuated Akt and rpS6 phosphorylation. Co-treatment with PI3K/Akt phosphorylation inhibitors increases the apoptosis rate at low concentrations of QUE (10μM) and 2-DG (2 mM).	[103]
Recombinant human PKM2 enzyme	9.24 µM	Inhibited PKM2 activity by 50%.	[100]

**Table 2:** Clinical trials evaluating the anti-cancer effect of QUE.

Clinical Study Title	Description	Dose, Via and Frequency of Administration	Benefits	Limitations	Ref
Sulindac and plant compounds in preventing colon cancer	Study the effectiveness of QUE among other compounds in preventing colon cancer. Estimated enrollment: Not specified Allocation: Randomized Primary Purpose: Prevention	Orally administered. 1 of 3 doses twice daily. For 6–10 weeks.	Determination of the lowest effective dose of QUE in modulating biomarkers of colon epithelial cell turnover, as an indication of colon cancer prevention.	No results published, although study completion date was 2006.	[113]
Pilot study evaluating broccoli sprouts in advanced pancreatic cancer	Administration of freeze-dried broccoli sprouts rich in QUE and sulforaphane in patients with advanced pancreatic ductal adenocarcinoma Estimated enrollment: 40 participants Allocation: Randomized Intervention Model: Parallel assignment	Orally administered. Capsules with broccoli sprout grain (90 mg sulforaphane daily + QUE-dose not specified). For 12 months.	Evaluation of cancer progress or regress in supplementation with capsules rich in QUE.	This study only declared sulforaphane concentration, but QUE content in the sprout was not specified. No results published, although study completion date was 2015.	[114]
Dietary Intervention in follicular lymphoma (Phase 2)	Assessment of the ability of grape juice (rich in QUE), among several dietary factors, to induce apoptosis, inhibit cell proliferation and modulate tumor cell infiltrate *in vivo*. Estimated enrollment: 45 participants Allocation: Non-Randomized Intervention Model: Single group assignment	Orally administered. Merlot grape juice 100%, 660 mL /495 mL every second day. For 16 weeks.	Determination of apoptosis of tumor cells as parameter of intervention efficacy.	The QUE content of the juice is unknown. The study completion date was 2009, however no results have been published yet.	[115]
Prostate cancer prevention with QUE and genistein	Evaluation of the effect of QUE or genistein supplementation, in comparison with placebo, against a PSA (prostate-specific antigen) increase. Estimated enrollment: 60 participants Allocation: Randomized Intervention Model: Crossover assignment	Orally administered. 500 mg QUE + vitamin C + folic acid + vitamin B3 daily. For 6 months.	Determination of QUE effect in PSA levels	This study evaluated a QUE supplement combined with other compounds that could act synergically or impact negatively over QUE effect, inducing side effects.	[116]
Effect of QUE on green tea polyphenol uptake in prostate tissue from patients with prostate cancer undergoing surgery (Phase 1)	Evaluation of the ability of QUE to enhance the uptake of green tea polyphenols in the prostate tissue of men taking green tea extract and undergoing radical prostatectomy. Evaluation of side effects of QUE in combination with green tea. Estimated enrollment: 31 participants Allocation: Randomized Intervention Model: Parallel assignment Primary Purpose: Prevention	Orally administered. Green tea polyphenol + QUE twice daily. For 3–6 weeks (before undergoing prostatectomy).	Determination of epigallocatechin gallate, epicatechin gallate and QUE concentration, and their methylated metabolites in prostate tissue and plasma. Determination of the extract and QUE on reducing the enzyme activity and protein and gene expression of catechol-O-methyltransferase (COMT), deoxyribonucleic methyltransferase 1 (DNMT1), and multidrug resistance transport protein 1 (MRP1) in prostate tissue.	The concentration of the extract and QUE used is not indicated.	[123]
Quercetin chemoprevention for squamous cell carcinoma in patients with fanconi anemia (FA) (Phase 2)	Efficacy of QUE in reducing buccal squamous cell carcinoma. Estimated enrollment: 55 participants Intervention Model: Single group assignment Primary Purpose: Prevention	Orally administered. Twice daily at an adjusted dose based on weight for a maximum total daily dose of 4000 mg/day. For 24 months	Evaluation of the efficacy of QUE in reducing buccal micronuclei and the need for potentially lethal treatment with chemotherapy and/or radiation therapy.	The primary outcome of this study is the reduction of buccal micronuclei in 45 post-hematopoietic cell transplantation (HCT) patients with FA. This is compared to only 10 patients FA patients without a history of HCT.	[124]
